# A Healthy Little Town!

**Published:** 1897-05-01

**Authors:** 


### Medical Progress and Hospital Clinics.

[*The Editor will be glad to receive offers of co-operation and contributions from members of the profession. All letters should be addressed to* The Editor, at the Office, 28 & 29, Southampton Street, Strand, London, W.C.]

### A HEALTHY LITTLE TOWN!

The medical officer of health for Harrow has issued a report which is remarkable in two respects.

First of all he is able to boast of a death rate of only 8·5 per 1,000; and we certainly congratulate him on having to deal with so healthy and select a set of people. Nothing, however, could more markedly illustrate the fallacies appertaining to crude returns than the fact that such a district should show a state of affairs which could only be maintained (except by migration), on condition that the inhabitants reached an average age of 117 years! Truly a step on the high road to immortality! No details are given as to the age constitution of the population, but it is quite clear that this and other influences causing a selection of lives are chiefly concerned in the production of such a low death rate, which may have but little to do with health conditions.

The second point in which this report is remarkable is the fact that its author draws attention to the ill effects upon the health of Harrow of the "absolutely inefficient" arrangements in London for the isolation of infectious diseases, and makes the following bold suggestion. "If," says he, "the richest city in the world will not make suitable provision, I think there ought to be some means by which her smaller and poorer neighbours should be able to recover from her for the expense and loss of life which her negligence has caused them." Here is, indeed, a new departure, and one which should make the lawyers rub their hands with glee. What a vista of litigation opens before us as we think of the "little bills" which sanitary authorities would send in to their neighbours for damage done after every epidemic. Fancy the feelings of some impecunious little district council on being asked to pay the expenses of an epidemic of milk typhoid in Marylebone or Islington, or on receiving a bill from the Asylums Board for an outbreak of small-pox due to their remissness in not having isolated some scabby tramp!

But is it true that all the virtue is on the side of Harrow? Let us compare this report with that for London for 1895—the last published. Take typhoid fever. The case rate in London was 0·8 per 1,000 living, against 0·59 in Harrow; not such a very striking difference, surely. Besides, London supplied hospital isolation for more than 25 per cent. of its cases; while not one of the Harrow cases seems to have been isolated in hospital.

The scarlet fever case rate was actually greater in Harrow than in London, and although a somewhat larger proportion of the cases were isolated, viz., 70 per cent. in Harrow against 57 per cent. in London, the difference in this respect is not very remarkable. Fortunately for Harrow it did not have much diphtheria, but of the cases that did occur not half as large a proportion were isolated in hospital as were in London, viz., only 16·6 per cent. against 34·4 per cent.

We have gone to the trouble to work this matter out and put it plainly before our readers, because the opinion is far too commonly held that London is a hot-bed of infection and a danger to all around. As a matter of fact diphtheria is the only notifiable disease which at present need give rise to serious anxiety in London; and even in regard to diphtheria, we must remember that if the authorities thought it desirable to isolate every individual case they could do so to-morrow by the simple expedient of refusing to admit so many cases of scarlet fever—a comparatively slight disorder. As a fact we believe that they are now admitting all the cases for which application is made, and they undoubtedly could admit many more. There are plenty of beds—it is a mere matter of choice how they shall be used.

